# Erhöhte Befundvollständigkeit und gesteigerte Zuweiserzufriedenheit bei strukturierter neurootologischer Befunderhebung in der interdisziplinären Schwindelabklärung

**DOI:** 10.1007/s00106-024-01464-5

**Published:** 2024-04-09

**Authors:** M. Lasrich, K. Helling, S. Strieth, K. Bahr-Hamm, T. J. Vogt, L. Fröhlich, T. Send, K. Hill, L. Nitsch, T. Rader, F. Bärhold, S. Becker, B. P. Ernst

**Affiliations:** 1 Klinik und Poliklinik für Hals-Nasen-Ohren-Heilkunde, Universitätsklinikum Bonn, Bonn, Deutschland; 2 Hals‑, Nasen‑, Ohrenklinik und Poliklinik – Plastische Operationen, Universitätsmedizin der Johannes Gutenberg-Universität Mainz, Mainz, Deutschland; 3 Klinik und Poliklinik für Neurologie, Universitätsklinikum Bonn, Bonn, Deutschland; 4 Klinik und Poliklinik für Hals-Nasen-Ohrenheilkunde, Abteilung Audiologie, LMU Klinikum der Ludwig-Maximilians-Universität München, München, Deutschland; 5 Nasen- und Ohrenheilkunde, Universitätsklinikum Tübingen, Universitätsklinik für Hals-, Tübingen, Deutschland; 6 Klinik für Hals‑, Nasen‑, Ohrenheilkunde, Universitätsklinikum Frankfurt, Theodor-Stern-Kai 7, 60590 Frankfurt, Deutschland

**Keywords:** Neurootologie, Arbeitsablauf, Datengenauigkeit, Audiometrie, Überweisung und Konsil, Neurotology, Workflow, Data accuracy, Audiometry, Referral and consultation

## Abstract

**Hintergrund:**

Befundberichte neurootologischer Funktionsdiagnostik im Rahmen der interdisziplinären Schwindelabklärung werden meist als Freitextbefunde („free text reports“, FTR) formuliert. Diese unterliegen häufig einer großen Variabilität, sodass hier Informationsverluste möglich sind. Ziel der vorliegenden Studie war es, die Befundvollständigkeit strukturierter Befunde („structured reports“, SR) und die Zuweiserzufriedenheit im Rahmen der neurootologischen Funktionsdiagnostik zu evaluieren.

**Material und Methoden:**

Retrospektiv wurden konsiliarisch durchgeführte neurootologische Funktionsdiagnostiken (*n* = 88) ausgewertet. Anhand der vorliegenden Rohdaten erfolgte mittels einer spezifischen Befunderhebungsmaske für neurootologische Funktionsdiagnostik die Erstellung korrespondierender SR zu den FTR aus der klinischen Routine. FTR und SR wurden auf Vollständigkeit und die Zufriedenheit der zuweisenden Ärzte (*n* = 8) mittels eines Fragebogens mit visueller Analogskala (VAS) untersucht.

**Ergebnisse:**

Im Vergleich zu den FTR zeigten die SR eine signifikant erhöhte Gesamtvollständigkeit (73,7 vs. 51,7 %; *p* < 0,001), insbesondere in Bezug auf die Anamnese (92,5 vs. 66,7 %; *p* < 0,001), Beschreibung von Vorbefunden (87,5 vs. 38 %; *p* < 0,001) und die neurootologische (33,5 vs. 26,7 %; *p* < 0,001) und audiometrische Funktionsdiagnostik (58 vs. 32,3 %; *p* < 0,001). Zudem zeigte sich mittels SR eine deutlich gesteigerte Zuweiserzufriedenheit (VAS 8,8 vs. 4,9; *p* < 0,001).

**Schlussfolgerung:**

Neurootologische SR ermöglichen eine deutlich gesteigerte Vollständigkeit der Befunde bei höherer Zufriedenheit der Zuweiser im Kontext der interdisziplinären Schwindelabklärung. Darüber hinaus eignen sich SR ideal zur wissenschaftlichen Datenanalyse, insbesondere im Rahmen von Big-Data-Analysen.

## Abstract (en)

**Background:**

Results of neurotological function diagnostics in the context of interdisciplinary vertigo assessment are usually formulated as free-text reports (FTR). These are often subject to high variability, which may lead to loss of information. The aim of the present study was to evaluate the completeness of structured reports (SR) and referrer satisfaction in the neurotological assessment of vertigo.

**Materials and methods:**

Neurotological function diagnostics performed as referrals (*n* = 88) were evaluated retrospectively. On the basis of the available raw data, SRs corresponding to FTRs from clinical routine were created by means of a specific SR template for neurotological function diagnostics. FTRs and SRs were evaluated for completeness and referring physician satisfaction (*n* = 8) using a visual analog scale (VAS) questionnaire.

**Results:**

Compared to FTRs, SRs showed significantly increased overall completeness (73.7% vs. 51.7%, *p* < 0.001), especially in terms of patient history (92.5% vs. 66.7%, *p* < 0.001), description of previous findings (87.5% vs. 38%, *p* < 0.001), and neurotological (33.5% vs. 26.7%, *p* < 0.001) and audiometric function diagnostics (58% vs. 32.3%, *p* < 0.001). In addition, SR showed significantly increased referring physician satisfaction (VAS 8.8 vs. 4.9, *p* < 0.001).

**Conclusion:**

Neurotological SRs enable a significantly increased report completeness with higher referrer satisfaction in the context of interdisciplinary assessment of vertigo. Furthermore, SRs are particularly suitable for scientific data analysis, especially in the context of big data analyses.

In den vergangenen Jahren wurde durch unterschiedliche Fachgesellschaften gezeigt, dass eine strukturierte Befunderhebung die Befundqualität, den Lernprozess von Weiterbildungsassistenten/-innen und die zeitliche Effizienz der Befunderstellung deutlich verbessert. Dabei eignet sich die strukturierte Befunderhebung insbesondere für Untersuchungen mit standardisierten Arbeitsabläufen. In diesem Beitrag wird daher die Erstellung einer strukturierten neurootologischen Befunderhebungsmaske und deren Validierung im Kontext der interdisziplinären Schwindelabklärung vorgestellt.

## Bedeutung fachspezifischer Diagnostik

Schwindel stellt einen häufigen Konsultationsgrund in verschiedenen Fachdisziplinen, u. a. der Hals‑, Nasen‑, Ohrenheilkunde (HNO), dar [19, 22]. In Notfallambulanzen geben bis zu 4 % der Patienten/-innen an, unter Schwindel zu leiden [12, 14]. Hierbei liegt eine Störung des Gleichgewichtsempfinden, der Wahrnehmung von Lage und Bewegung, der körperlichen Integrität beim Stehen und Gehen sowie der visuellen, labyrinthären und propriozeptiven Integrität vor [1, 3]. Patienten/-innen beschreiben Schwindel als Unwohlsein und Unsicherheit mit verwackeltem Sehen [20]. Ein Großteil der Betroffenen leidet unter einer nicht lebensbedrohlichen Ursache. Hierbei sind bei den nicht peripher-vestibulären Schwindelbeschwerden insbesondere neurologische, psychosomatische und kardiovaskuläre Ursachen sowie orthopädische Beschwerden häufig [15]. Bei den peripher-vestibulären Ursachen dominieren der benigne paroxysmale Lagerungsschwindel, die akute unilaterale Vestibulopathie/Neuropathie und der M. Menière [3]. Ferner bestehen Schwindelbeschwerden häufig bei Patienten/-innen vor Cochleaimplantatversorgung als Ausdruck der hier zugrunde liegenden vestibulocochleären Störung oder können postoperativ nach Implantation auftreten oder exazerbieren [17, 24]. Demgegenüber liegt bei bis zu 5–12 % der Patienten/-innen mit akutem vestibulären Syndrom und bei bis zu 25 % der Vorstellungen in neurologischen Notaufnahmen eine potenziell lebensbedrohliche zerebrovaskuläre oder kardiovaskuläre Erkrankung vor [25]. Diese Ursachen gilt es aufgrund des teils engen therapeutischen Zeitfensters schnell und sicher zu identifizieren. Eine häufige benigne Ursache stellt darüber hinaus die vestibuläre Migräne dar [3].

Wegen der häufig multifaktoriellen Genese kann eine genaue Abklärung äußerst komplex sein und erfordert oftmals ein interdisziplinäres Vorgehen, sowohl im ambulanten als auch im stationären Bereich. Eine exakte Anamnese einschließlich aller spezifischen Symptome und eine umfassende fachspezifische Diagnostik sind dabei unabdingbar. Zudem haben bei neu aufgetretenen Beschwerden die genaue Anamnese einschließlich der neurootologischen Funktionsdiagnostik eine höhere Sensitivität als Magnetresonanztomographien allein [27]. Aufgrund der meist interdisziplinären Schwindelabklärung ist eine hohe Befundqualität essenziell, um die erhobenen Untersuchungsbefunde und deren Interpretation für die zuweisenden Kollegen/-innen nachvollziehbar zu übermitteln. Nur so lassen sich Informationsverluste, redundante Untersuchungsschritte und Verzögerungen bei der Diagnosefindung und Therapieeinleitung vermeiden [18].

Trotz der Vereinheitlichung der Facharztausbildung durch Definition von Standards sowie der verfügbaren hochqualitativen Leitlinien wurden die Form sowie die Inhalte der Befunddokumentation als einer der Hauptgründe für Informationsverluste und Unzufriedenheit bei zuweisenden Ärzten/-innen identifiziert [6]. Eine mögliche Lösung dafür stellt die Implementierung strukturierter Befunde („structured reports“, SR) dar. In den vergangenen Jahren wurden SR durch verschiedene Fachgesellschaften unterstützt, da von ihnen eine signifikante Verbesserung der Befundqualität im Vergleich zu konventionellen Freitextbefunden („free text reports“, FTR) ausgeht [11, 21, 26]. Darüber hinaus können SR aufgrund ihrer standardisierten Struktur besser für hochwertige wissenschaftliche Datenanalysen verwendet werden [23]. Mehrere Studien ergaben für den Einsatz von SR in der HNO-Heilkunde, dass es zu einer Verbesserung der Befundqualität, der Interrater-Reliabilität, der zeitlichen Effizienz sowie der Ausbildung kommt [6, 7, 9, 10]. Zugleich verbessern SR die interdisziplinäre Zusammenarbeit, [2, 8, 16, 29].

Das Ziel der vorliegenden Studie war es, eine strukturierte neurootologische Befunderhebungsmaske zu implementieren und diese im Kontext der interdisziplinären Schwindelabklärung auf Anwendbarkeit, Vollständigkeit und Zuweiserzufriedenheit im Vergleich zu FTR zu bewerten.

## Methodik

### Studiendesign

Es erfolgte die im Zeitraum 2020–2021 die retrospektive Identifikation von 100 konsekutiven Patienten/-innen, die konsiliarisch in der Klinik und Poliklinik für Hals-Nasen-Ohren-Heilkunde des Universitätsklinikums Bonn zur neurootologischen Funktionsdiagnostik im Rahmen einer interdisziplinären Schwindelabklärung vorgestellt wurden. Die digitalen Patientenakten wurden hinsichtlich der Verfügbarkeit von Anamnese sowie Rohdaten der neurootologischen und audiometrischen Diagnostik untersucht. Die im regulären Klinikbetrieb erhobenen FTR wurden als Kontrollgruppe verwendet.

### Berechnung der Stichprobengröße

Die Anzahl der benötigten Patienten/-innen wurde, analog zu bereits publizierten Studien, auf Grundlage der erwarteten Effektgröße berechnet, bei der der Prozentsatz von FTR mit einer Vollständigkeit von 80 % oder mehr mit SR verglichen wird [5, 6]. Aufgrund mangelnder Daten zur Befundqualität von neurootologischer Funktionsdiagnostik wurde geschätzt, dass 65 % der FTR eine Vollständigkeit von 80 % oder mehr aufweisen würden. Ferner haben die Autoren eine Erhöhung dieses Anteils auf 85 % bei SR angenommen. Die Power wurde auf 80 % und das Signifikanzniveau auf α = 0,05 festgesetzt. Anhand dieser Parameter wurde die Mindestanzahl der Patienten/-innen ermittelt, die sich auf *n* = 144 (72 Befunde in jeder Gruppe) belief.

### Strukturierte Befunderhebung

**Abb. 1:**
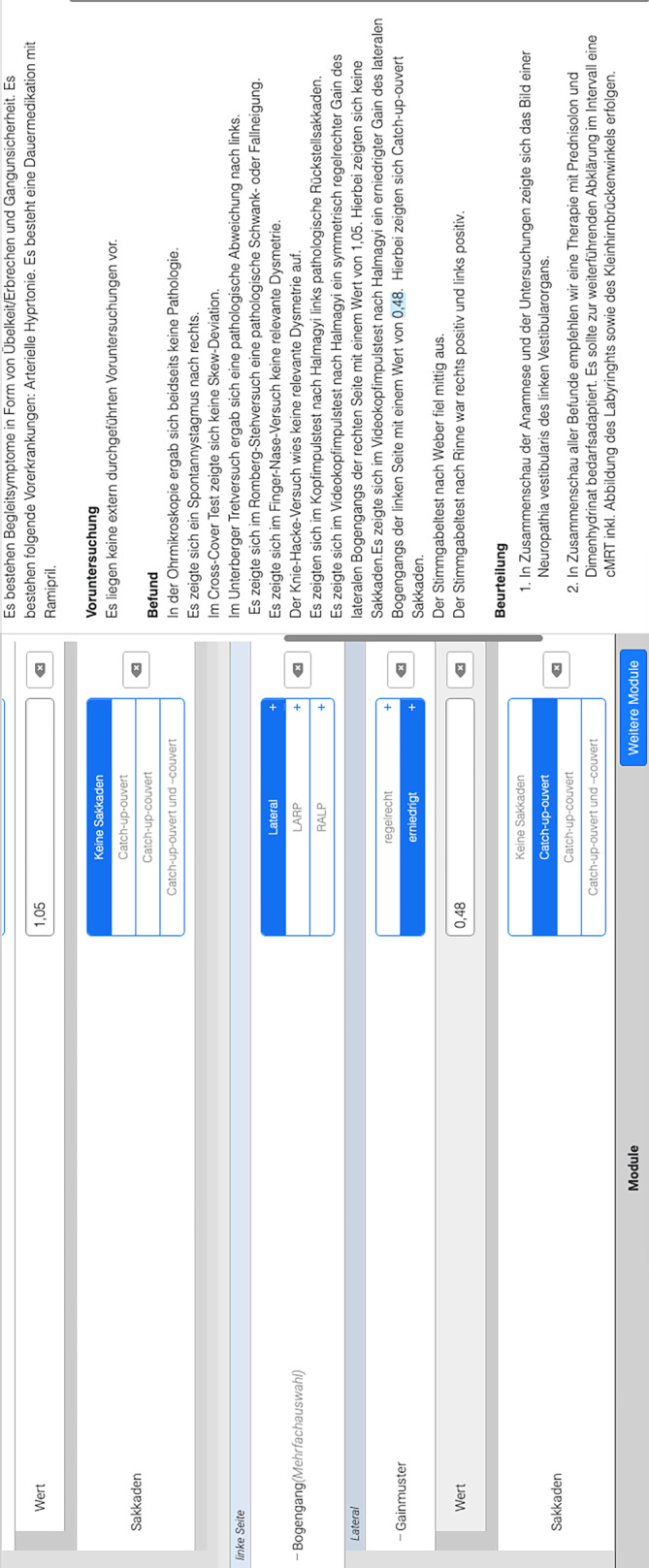
Screenshot des Entscheidungsbaums in der strukturierten neurootologischen Befunderhebungssoftware. Erläuterung s. Text. Bericht über eine Neuropathia vestibularis links. Angaben zu Anamnese, Voruntersuchungen, körperlicher Untersuchung, Funktionsdiagnostik sowie Diagnose und Therapie *links*. Durch Befunderhebungssoftware in Echtzeit erstellter vollständiger Befundtext *rechts*

Zur strukturierten Befunderhebung wurde eine webbasierte Software (Smart Reporting GmbH, München, Deutschland, http://www.smart-reporting.com/de) verwendet, um eine spezielle Befunderhebungsmaske für die strukturierte neurootologische Befunderhebung zu erstellen (Abb. 1). Abgebildet in Abb. 1 ist ein beispielhafter Bericht über eine Neuropathia vestibularis links. Auf der linken Seite werden Angaben zu Anamnese, Voruntersuchungen, körperlicher Untersuchung sowie zur neurootologischen und audiometrischen Funktionsdiagnostik sowie zur abgeleiteten Diagnose und Therapie abgefragt. Die Befunderhebungssoftware erstellt in Echtzeit auf der rechten Seite einen vollständigen, semantisch korrekten und strukturierten Befundtext.

Die Vorlage wurde im Konsensverfahren von 3 erfahrenen Fachärzten/-innen für Hals‑, Nasen‑, Ohrenheilkunde sowie 2 Audiologen/-innen erstellt. Die Befunderhebungsmaske war derart konzipiert, dass mit ihr die neurootologische Anamnese, Vorbefunde, die klinische Untersuchung einschließlich Ohrmikroskopie, vestibulospinaler Reflexe und Koordination sowie alle am Universitätsklinikum Bonn etablierten neurootologischen (kalorische Prüfung, Videonystagmographie, Videokopfimpulstest und zervikale vestibulär evozierte Potenziale) und audiologischen Funktionstests erfasst werden können. In der Folge können mittels Vorauswahl, aber auch durch Möglichkeit zur Nutzung von Freitextfeldern alle gängigen neurootologischen Krankheitsbilder abdeckt werden. Durch anklickbare Entscheidungsbäume werden hier anhand zuvor definierter Textbausteine vollständige semantische Sätze generiert und zu einem stets einheitlichen Befundaufbau zusammengesetzt. Um ein Maximum an Flexibilität zu gewährleisten, haben Anwender/-innen grundsätzlich die Möglichkeit, den strukturierten Befund um Freitextelemente zur erweitern oder zu präzisieren. Darüber hinaus können spezifische Anleitungen und Tutorials der integrierten Funktionstests einschließlich Beispielbefunden in die Vorlage integriert werden. Dies reduziert die Notwendigkeit zur Konsultation weiterführender medizinischer Literatur während der Befunderstellung. Nach Etablierung der strukturierten neurootologischen Befunderhebungsmaske erfolgte anhand der vorliegenden Anamnese sowie der Untersuchungsrohdaten die Erstellung korrespondierender SR zu den FTR aus der klinischen Routineversorgung.

### Befundevaluation

Die anonymisierten Berichte wurden im Hinblick auf Vollständigkeit der Anamnese, der Dokumentation etwaiger Vorbefunde, des ohrmikroskopischen Befundes sowie der neurootologischen und audiometrischen Diagnostik erfasst und bewertet. Für die Bewertung wurde, analog zu vorherigen Publikationen, eine von 3 HNO-Fachärzten/-innen und 2 Audiologen/-innen speziell entwickelte Checkliste verwendet [6, 7, 9]. Aufgrund des retrospektiven Charakters der Studie und der gepaarten Stichprobenanalyse wurde hier, entgegen der Literatur, die zu einer ökonomischen Anordnung neurootologischer Funktionsprüfungen rät, als Goldstandard ein Maximum an Informationsausbeute gewählt, um eine möglichst gute Differenzierung der Vollständigkeit zwischen FTR und SR zu gewährleisten [4].

Zusätzlich wurde, ebenfalls durch 3 HNO-Fachärzten/-innen und 2 Audiologen/-innen, ein Fragebogen für die zuweisenden Ärzte/-innen erstellt. Anhand einer 10-stufigen visuellen Analogskala (VAS, 10: völlige Zustimmung, 0: völlige Ablehnung) wurden die zuweisenden Ärzte/-innen (*n* = 8) nach der Struktur und dem Layout des Befundes (Q1), der Vollständigkeit der Anamnese (Q2), der Voruntersuchungen (Q3), des ohrmikroskopischen Befundes (Q4), der neurootologischen (Q5) und audiometrischen Funktionsdiagnostik (Q6), der Verständlichkeit des Gesamtbefundes (Q7) sowie zur Beantwortung der Fragestellung (Q8) und der Nachvollziehbarkeit von Diagnose und Therapieempfehlung (Q9) befragt. Den zuweisenden Ärzten/-innen wurden anonymisiert jeweils 5 korrespondierende SR und 5 FTR vorgelegt, anhand derer der Fragebogen zur Zuweiserzufriedenheit zu beantworten war.

### Statistische Analyse

Die Daten werden als Mittelwert ± SD („standard deviation“, Standardabweichung) angegeben. Die erfassten Variablen waren gemäß dem Shapiro-Wilk-Test normalverteilt. Der gepaarte t‑Test wurde zum Vergleich der Gesamtvollständigkeit und zur Evaluation der Benutzerzufriedenheit verwendet. Ein *p*-Wert von weniger als 0,05 wurde als statistisch signifikant angesehen. Alle statistischen Analysen wurden mit GraphPad Prism 9.5.1 (Graphpad Software LLC., San Diego, CA, USA) und Microsoft Excel 2019 (Microsoft Corporation, Redmond, WA, USA) durchgeführt.

## Ergebnisse

### Befundanalyse

**Abb. 2:**
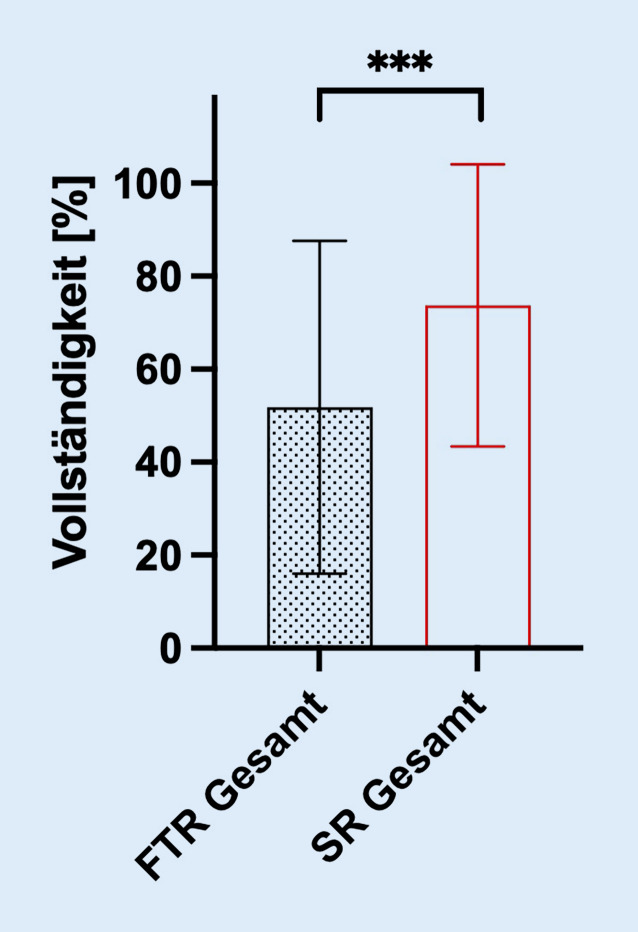
Ergebnisse der Gesamtbefundanalyse. Signifikant höhere Befundvollständigkeit der strukturierten Befunde (SR) als der konventionellen Freitextbefunde (FTR) in der Gesamtanalyse. *** *p* < 0,001

Insgesamt konnten 176 Befundberichte (jeweils *n* = 88 für FTR und SR) in die Analyse eingeschlossen werden. Bei insgesamt 24 Befundberichten (jeweils *n* = 12 für FTR und SR) waren in der digitalen Patientenakte keine vollständige Anamnese bzw. keine vollständigen Rohdaten der apparativen Diagnostik vorhanden. SR wiesen eine signifikant höhere Gesamtvollständigkeit auf (73,7 vs. 51,7 %; *p* < 0,0001; Abb. 2).

Im Einzelnen erzielten SR höhere Bewertungen der Vollständigkeit in Bezug auf die Anamnese (92,5 vs. 66,7 %; *p* < 0,0001), die Zusammenfassung etwaiger Voruntersuchungen (87,5 vs. 38 %; *p* < 0,0001) sowie die neurootologische (33,5 vs. 26,7 %; *p* < 0,0001) und audiometrische Funktionsdiagnostik (58 vs. 32,3 %; *p* < 0,0001). Die Vollständigkeit des ohrmikroskopischen Befundes war unabhängig von der Befundungsmodalität sehr hoch (97 vs. 95 %; *p* = 0,1583, Abb. 3).

**Abb. 3:**
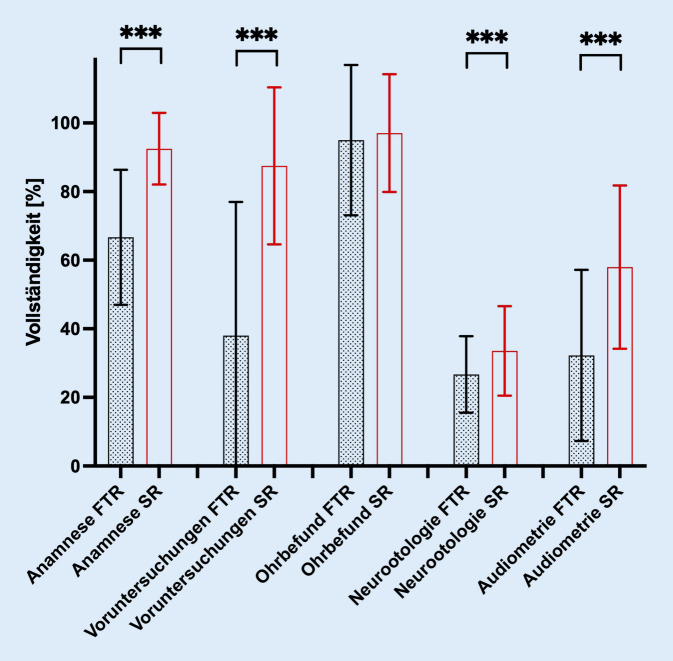
Ergebnisse der Untergruppenanalyse. Signifikant höhere Vollständigkeit der strukturierten Befunde (SR) in der detaillierten Analyse im Vergleich zu konventionellen Freitextbefunden (FTR) für die Anamnese, die Voruntersuchungen sowie bei der neurootologischen und audiometrischen Diagnostik. *** *p* < 0,001

### Zuweiserzufriedenheit

**Abb. 4:**
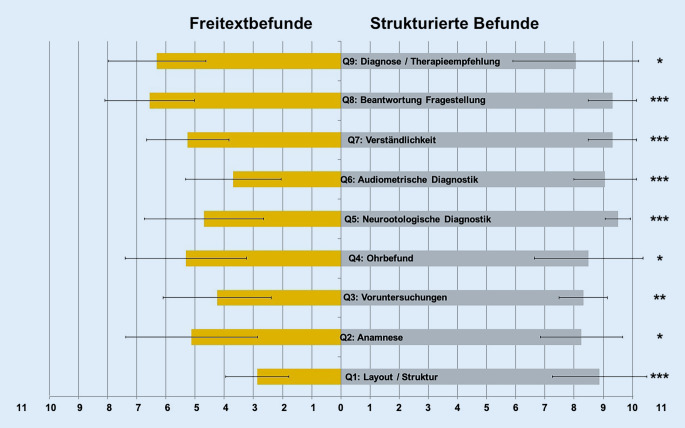
Visuelle Analogskala (VAS) der Fragebogenergebnisse zur Zufriedenheit der zuweisenden Ärzte/-innen. Gemäß visueller Analogskala (VAS; 10: völlige Zustimmung, 0: völlige Ablehnung) bei strukturierten Befundberichten (SR, *rechte* *Seite, graue Balken*) im Vergleich zu konventionellen Freitextbefunden (FTR, *linke Seite, orangefarbene Balken*) übersichtlicheres Layout und bessere Strukturierung (Q1), signifikant bessere Vollständigkeit von Anamnese (Q2), Zusammenfassung der Voruntersuchungen (Q3), ohrmikroskopischem (Q4) Befund sowie der neurootologischen (Q5) und audiometrischen Voruntersuchungen (Q6). SR durch Zuweiser/-innen als verständlicher empfunden (Q7). Eindeutigere Beantwortung der Fragestellung mittels SR (Q8) und nachvollziehbarere Diagnose und Therapieempfehlung (Q9). * *p* < 0,05, ** *p* < 0,01, *** *p* < 0,001

Die Bewertung der VAS-basierten Fragebögen zeigte eine sehr hohe Zufriedenheit der zuweisenden Ärzte/-innen für die neurootologischen SR im Vergleich zu den FTR (8,8 vs. 4,9; *p* < 0,0001). Im Einzelnen bevorzugten die Zuweiser/-innen die SR hinsichtlich ihrer Struktur und ihres Layouts (Q1, 8,9 vs. 2,9; *p* < 0,001), der Vollständigkeit der Anamnese (Q2, 8,25 vs. 5,1; *p* = 0,012), der Zusammenfassung der Voruntersuchungen (Q3, 8,3 vs. 4,3; *p* = 0,0026), des ohrmikroskopischen Befundes (Q4, 8,5 vs. 5,3; *p* = 0,0218) sowie der neurootologischen (Q5, 9,5 vs. 4,7; *p* = 0,0007) und audiometrischen Funktionsdiagnostik (Q6, 9,1 vs. 3,7; *p* = 0,0001). Darüber hinaus wurden die SR als verständlicher (Q7, 9,3 vs. 5,3; *p* = 0,0005), die Beantwortung der Fragestellung als eindeutiger (Q8, 9,3 vs. 6,6; *p* = 0,0007) und die Diagnose und Therapieempfehlung als nachvollziehbarer (Q9, 8,1 vs. 6,3; *p* = 0,0358) bewertet (Abb. 4).

## Diskussion

Neben einer eingehenden körperlichen Untersuchung einschließlich der HNO-ärztlichen Spiegeluntersuchung hat die neurootologische Funktionsdiagnostik einen zentralen Stellenwert bei der interdisziplinären Abklärung von Schwindelbeschwerden aller Art. Aufgrund der Komplexität des Erkrankungsspektrums und des interdisziplinären Ansatzes ist eine exakte Befundberichterstellung essenziell, um die höchsten Standards in Diagnostik und Therapie zu gewährleisten. Ziel der vorliegenden Studie war es daher, eine strukturierte Befunderhebungsmaske für die Dokumentation neurootologischer Funktionsdiagnostik zu entwickeln und diese im Kontext der interdisziplinären Schwindelabklärung auf Alltagstauglichkeit, Befundvollständigkeit und Zufriedenheit der zuweisenden Ärzte/-innen zu evaluieren. Soweit den Autoren bekannt ist, existierten bisher keine Studien zur Anwendung von SR in der Neurootologie.

### Einfluss der Befunderhebungsmaske

Die Entwicklung und orientierende Überprüfung der Befunderhebungsmaske erfolgte im Konsensverfahren durch 3 Fachärzte/-innen für Hals‑, Nasen‑, Ohrenheilkunde sowie 2 Audiologen/-innen mit großer Expertise in der neurootologischen Funktionsdiagnostik. Die erhobenen Daten belegen, dass die Anwendung von SR in der neurootologischen Funktionsdiagnostik zu einer signifikant erhöhten Befundvollständigkeit führt. Die Analyse der Zufriedenheit der zuweisenden Ärzte/-innen im interdisziplinären Setting zeigte eine klare Präferenz für SR. Diese Ergebnisse stimmen mit früheren Veröffentlichungen für andere diagnostische Modalitäten überein, in denen die Verwendung einer strukturierten Befundung mit einer besseren Befundqualität für ein breites Spektrum an diagnostischen Modalitäten korreliert werden konnte [11, 21, 26, 30].

### Neurootologische Funktionsdiagnostik

Auch wenn die vorliegenden Daten zeigen, dass die Vollständigkeit der Befunde signifikant gesteigert werden konnte, muss auf die weiterhin unterdurchschnittliche Vollständigkeit der neurootologischen Funktionsdiagnostik innerhalb der SR, dem Kernaspekt der vorliegenden Studie, hingewiesen werden. Während die sonstigen Teilaspekte der SR wie Anamnese, Voruntersuchungen, audiometrische Diagnostik und Ohrmikroskopie eine mittlere Vollständigkeit von 83,8 % (vs. 58 %; *p* < 0,001) aufwiesen, verblieb die Vollständigkeit der neurootologischen Funktionsdiagnostik bei lediglich 33,5 % (vs. 26,7 % bei FTR; *p* < 0,001; Abb. 3). Diesbezüglich müssen mehrere Teilaspekte bedacht werden. Zum einen wurden die strukturierte Befunderhebungsmaske sowie die Checkliste zur Beurteilung der Befundvollständigkeit unter Berücksichtigung der Vorgaben der S2k-Leitlinie „Vestibuläre Störungen“ und entsprechend der diagnostischen Möglichkeiten der Klinik und Poliklinik für Hals-Nasen-Ohren-Heilkunde zum Studienzeitpunkt 2023 entwickelt [3]. Folglich waren im retrospektiven Beobachtungszeitraum zwischen 2020 und 2022 Teilaspekte der neurootologischen Funktionsdiagnostik, wie etwa zervikale und okuläre vestibulär evozierte myogene Potenziale oder auch der triplanare Videokopfimpulstest nicht durchgängig etabliert bzw. wartungsbedingt nicht zu jedem Zeitpunkt verfügbar. Da die strukturierte Befunderhebungsmaske und in der Folge auch die Checkliste zur Befundbewertung stets alle Teilaspekte der neurootologischen Diagnostik abfragt, resultiert hieraus a priori eine entsprechend reduzierte Vollständigkeit, sowohl für SR als auch für FTR, in dem jeweiligen Teilaspekt. Zum anderen muss an dieser Stelle ein Fortsetzungsfehler bedacht werden. Wurden bei der konsiliarisch durchgeführten neurootologischen Funktionsdiagnostik Teilaspekte nicht durchgeführt oder waren zum Untersuchungszeitpunkt aus technischen Gründen nicht verfügbar, so standen in einem solchen Fall keine Rohdaten bei der Erstellung der korrespondierenden SR zur Verfügung. Insofern ist hier auch eine Übertragung der unterdurchschnittlichen Vollständigkeit des neurootologischen Teilgebiets in den FTR auf die SR möglich. Dennoch resultiert aus der Verwendung von SR, selbst unter Berücksichtigung der entsprechenden, teils unvollständigen Rohdaten, eine signifikant gesteigerte Befundvollständigkeit.

### Testökonomie

Die neurootologische Funktionsdiagnostik als Teilaspekt der Schwindelabklärung ist ein komplexes, interdisziplinäres Feld, das insbesondere für Weiterbildungsassistenten/-innen aller Fachgebiete häufig eine Herausforderung darstellt. Dies beginnt bei der Formulierung der Fragestellung in der konsiliarischen Anforderung bezüglich der Mitbeurteilung und Diagnostik, setzt sich in der Umsetzung der spezifisch notwendigen Diagnostik und Ausformulierung der Befundberichte fort und endet bei der führenden Fachdisziplin mit der Würdigung bzw. der Umsetzung des Befundberichts. Dabei wird in der Literatur insbesondere darauf hingewiesen, dass insbesondere die Testökonomie von großer Bedeutung ist [4]. Dabei kann eine spezifische, an die Beschwerden der Patient/-innen angepassten Auswahl der neurootologischen Tests zur Diagnosefindung beitragen und helfen, die Frustration, sowohl bei Patienten/-innen als auch Untersucher/-innen, zu reduzieren und damit die Compliance zu verbessern. In diesem Szenario könnte die SR eine zentrale Hilfestellung darstellen, in dem spezifische Symptome mit dezidierten Funktionsdiagnostiken assoziiert werden. Dies könnte zu einer gesteigerten Effizienz und zum Einsparen der meist knapp vorhandenen Ressourcen beitragen. Weiterbildungsassistenten/-innen fällt es regelhaft schwer, die Ergebnisse neurootologischer Funktionsdiagnostik adäquat zu interpretieren und zu beschreiben, was zu großer Frustration führen kann. Dies spiegelt sich auch in einem sinkenden Interesse an einer neurootologischen Spezialisierung wider [13]. Golub et al. konnten in einer Befragung von 1364 Weiterbildungsassistenten/-innen in den USA zeigen, dass etwa 14 % im zweiten Weiterbildungsjahr ein Interesse an einer neurootologischen Spezialisierung angaben, während dies nur bei etwa 7 % im fünften Weiterbildungsjahr der Fall war (−53 %). Als Konsequenz kann es zu einer unzureichenden Qualität der Befundberichte kommen, die möglicherweise wiederum für fachfremde Ärzte/-innen dann unzureichend nachvollziehbar sind. Dies steht in großem Widerspruch zu den Anforderungen, die an die Befundung gestellt werden [30]. Jedem Bericht über ein medizinisches Diagnoseverfahren kommt große Rolle zu, da der Bericht den Inhalt und die Schlussfolgerungen interpersonell und interdisziplinär transportiert [6]. Zudem weisen FTR eine deutlich niedrigere Interrater-Reliabilität und somit eine höhere Befundvariabilität auf als SR [5]. Durch Informationsverluste aufgrund von unvollständigen oder unverständlichen Befundberichten kann es, neben der Frustration der Zuweiser/-innen, potenziell zu redundanten, zeit- und kostenintensiven Folgeuntersuchungen kommen. Zudem besteht die Gefahr verzögerter Diagnosestellungen und Therapieeinleitungen, möglicherweise mit rechtlichen Konsequenzen [5]. Dies kann durch eine unzureichende Reproduzierbarkeit der Befunddokumentation verstärkt werden. In diesem Szenario kann die Verwendung von SR zur Ausbildung beitragen, indem sie unerfahrene Weiterbildungsassistenten-/innen durch die Diagnostik und deren Befundung führt, zentrale Aspekte aufzeigt und somit die Interrater-Reliabilität nachhaltig verbessert [5]. Durch vorherige Studien wurde gezeigt, dass die Verwendung von SR mit einer deutlichen Reduktion inadäquat untersuchter pathologischer Veränderungen einhergeht [28]. Darüber hinaus konnte am Beispiel der Kopf-Hals-Sonographie ein additiver, longitudinaler Lerneffekt durch die konsequente Anwendung von SR in der Facharztweiterbildung nachgewiesen werden [9]. Folglich ist ein solcher Effekt auch durch die Implementierung der SR im Bereich der Neurootologie anzunehmen. Darüber hinaus unterstützt die Anwendung von SR die behandelnden Ärzte/-innen, indem sie durch die systematische Vorgehensweise hilft, die unterschiedlichen schwindelspezifischen Kausalitäten effektiv zu identifizieren respektive diese auszuschließen. Durch die hinterlegten klinischen Behandlungspfade können entsprechend relevante Differenzialdiagnosen aufgezeigt und adäquat dokumentiert werden. Hierdurch wird eine leitliniengetreue Diagnostik und Therapie und in der Folge die evidenzbasierte Medizin nachhaltig unterstützt. Somit können Schwindelbeschwerden bei Patienten/-innen effektiver abgeklärt und therapiert werden. Neben der Diagnosefindung und Therapieeinleitung ermöglichen SR aber auch ein effizientes Monitoring des Krankheitsverlaufs. Aufgrund der stets einheitlichen Darstellung der Befunde können krankheitsspezifische Veränderungen, insbesondere für beteiligte Berufsgruppen wie beispielswiese Physiotherapeuten, leichter erkannt und somit Therapien besser angepasst werden. Dies führt nicht nur zu einer verbesserten Kommunikation zwischen den verschiedenen ärztlichen Fachgebieten, sondern auch mit den anderen beteiligten Berufsgruppen bzw. Rehabilitationseinrichtungen.

Ob die höhere Befundvollständigkeit und die verbesserte Zuweiserzufriedenheit der SR tatsächlich mit einer effizienteren Diagnosestellung und Therapieeinleitung sowie einer verbesserten interdisziplinären Kommunikation verbunden ist, müssen zukünftige Studien klären.

## Fazit für die Praxis

- Eine strukturierte Befunderhebung ist im klinischen Alltag dazu geeignet, die Vollständigkeit der neurootologischen Funktionsdiagnostik signifikant zu steigern.
- Die strukturierte neurootologische Befunderhebung unterstützt die interdisziplinäre Schwindelabklärung und verbessert die Zufriedenheit externer Zuweiser/-innen.
- Mittels einer strukturierten Befunderhebung könnte potenziell die interdisziplinäre Kommunikation verbessert und damit Informationsverluste, redundante Folgeuntersuchungen sowie Verzögerungen bei Diagnosestellung und Therapieeinleitung vermieden werden.
